# Relationship Between Immunosuppressive Therapy and the Development of Infectious Complications Among Patients with Anti-neutrophil Cytoplasmic Antibody-associated Vasculitis: A Single-center, Retrospective Observational Study

**DOI:** 10.7759/cureus.5676

**Published:** 2019-09-16

**Authors:** Makoto Harada, Wataru Ishii, Takeshi Masubuchi, Tohru Ichikawa, Mamoru Kobayashi

**Affiliations:** 1 Department of Nephrology, Shinshu University, Matsumoto, JPN; 2 Department of Rheumatology, Nagano Red Cross Hospital, Nagano, JPN; 3 Department of Respiratory Medicine, Nagano Red Cross Hospital, Nagano, JPN; 4 Department of Nephrology, Nagano Red Cross Hospital, Nagano, JPN

**Keywords:** anti-neutrophil cytoplasmic antibody-associated vasculitis, cyclophosphamide, infection, methylprednisolone pulse

## Abstract

Introduction

Infectious complications are the leading cause of death in patients with anti-neutrophil cytoplasmic antibody-associated vasculitis (AAV). However, the relationship between initial immunosuppressive therapy and the development of infectious complications and the details of infectious complications among patients with AAV are uncertain. We thus aimed to determine the association between initial immunosuppressive therapy and infectious complications.

Material and methods

Forty-seven patients with newly diagnosed AAV were enrolled in this retrospective observational study (patients with eosinophilic granulomatous polyangiitis were excluded). We statistically determined the association between types of initial immunosuppressive therapy (methylprednisolone pulse and/or cyclophosphamide therapy) and the development of infectious complications. In addition, we investigated the causes and timing of the onset of infectious complications.

Results

Twenty-one (21; 44.7%) patients required antibiotic, antimycotic, or antiviral therapy because of the development of infectious complications. Multiple logistic regression analyses adjusted for age and sex revealed that methylprednisolone pulse and cyclophosphamide therapy were significantly associated with the development of infectious complications (odds ratio (OR) 4.85, 95% confidence interval (CI) 1.09-21.5, p = 0.038; OR 5.32, 95% CI 1.28-22.2, p = 0.022, respectively). Bacterial pneumonia and sepsis occurred in 10 (47.6%) and 6 (28.6%) patients, respectively. Almost half of these infectious complications, including fungal infection, developed within six months from the start of initial treatment.

Conclusion

Among patients with AAV, methylprednisolone pulse and cyclophosphamide therapy may increase the risk of developing infectious complications, such as pneumonia and sepsis, including fungal infection, particularly within six months from the initiation of treatment.

## Introduction

Anti-neutrophil cytoplasmic antibody (ANCA)-associated vasculitis (AAV) is a systemic small-vessel vasculitis that is positive for ANCA [1-2]. Systemic AAV is classified into three main categories: microscopic polyangiitis (MPA), granulomatosis with polyangiitis (GPA), and eosinophilic granulomatosis with polyangiitis (EGPA) [1,3]. MPA and GPA often cause severe lung and kidney injuries and can lead to interstitial pneumonia, alveolar hemorrhage, and pulmonary multiple nodular lesions, resulting in respiratory failure [3-7]. MPA and GPA cause necrotizing crescentic glomerulonephritis with pauci-immunity that can result in renal failure [3-7]. Immunosuppressive agents, such as steroids, cyclophosphamide, methotrexate, and rituximab, are used for initial and induction therapy [2,5-6]. In Japan, most patients with AAV are treated with steroids and approximately 30% are treated with cyclophosphamide as an initial therapy [2,4]. Rituximab therapy for AAV is being gradually adopted in Japan. Because the main cause of death in patients with AAV is infectious complications, it is important to evaluate patients with a high risk of infectious complications carefully. It is thought that immunosuppressive therapy influences the development of infectious complications. However, the relationship between the type of initial immunosuppressive therapy and the development of infectious complications among patients with AAV and details concerning infectious complications that develop during the therapeutic course of patients with AAV are unknown. Few studies have investigated the risk factors for developing infectious complications [8]. In this study, we aimed to determine the association between the types of initial immunosuppressive therapy and the development of infectious complications. In particular, we aimed to evaluate the association between methylprednisolone and/or cyclophosphamide therapy (that is mainly recommended in Japanese patients with AAV) and the development of infectious complications. In addition, we investigated the details of infectious complications, the timing of onset, and types.

## Materials and methods

Patient population and study design

This was a retrospective observational study conducted in Nagano Red Cross Hospital from January 2010 to December 2017. Of all the patients with AAV, those with EGPA were excluded. The reason why we excluded patients with EGPA in the current study is as follows: the frequency of severe kidney injury is different between EGPA and other types of AAV (MPA and GPA). Almost 60% to 80% of patients with MPA or GPA were complicated with rapid progressive glomerulonephritis while approximately 20% of patients with EGPA were complicated with rapid progressive glomerulonephritis [1]. In addition, the type and manifestation of lung involvement is also different between EGPA and other types of AAV (MPA and GPA). Almost 60% to 80% of patients with MPA or GPA develop lung lesion. The representative lung involvement is alveolar hemorrhage, which causes severe respiratory failure. However, although all cases are complicated by asthma, almost half of patients with EGPA develop transient patchy infiltration or eosinophil pleural effusion [1]. In general, EGPA is often controlled with glucocorticoid therapy [1], therefore, it is possible that the strength of immunosuppressive therapy in patients with EGPA is weaker than that in patients with MPA or GPA. Thereafter, 57 patients were enrolled, and 10 were excluded (nine patients had been treated before admission and one case of AAV was thought to be caused by thiamazole therapy). Finally, 47 patients newly diagnosed with AAV were enrolled, of which four, 18, 18, and seven patients were categorized as GPA, MPA, renal-limited vasculitis (RLV), and unclassifiable, respectively. The study protocol was approved by the institutional review board of the ethical committee at Nagano Red Cross Hospital and was conducted in accordance with the principles of the Declaration of Helsinki as revised in 2013. Clinical data were collected from patients’ medical records. Laboratory data (type of ANCA, white blood cell (WBC) count, hemoglobin, albumin, blood urea nitrogen, creatinine, c-reactive protein, and urinary abnormalities) at the time of hospital admission were collected.

Definitions

AAV was defined according to the results of the algorithm suggested by Watts et al. [7]. We categorized each AAV case as GPA, MPA, RLV, and unclassifiable. Patients with drug-induced vasculitis were excluded. Diabetes mellitus was defined as a high level of HbA1c (>6.5%) and/or prescription of insulin or hypoglycemic agents. A history of hypertension was defined as a prescription of anti-hypertensive drugs. Interstitial pneumonia was defined as presenting with bilateral interstitial lesions on computed tomographic (CT) images. Alveolar hemorrhage was defined as presenting with hemoptysis and lung abnormality on computed tomography that corresponded to hemorrhage. Neurological symptoms were defined as numbness and muscle weakness. The maximum dose of prednisolone (PSL) was adjusted according to the ideal body weight. In a previous Japanese nationwide study, rapid PSL reduction was defined when the necessary daily PSL dose decreased to <20 mg/day within eight weeks [4]. Methylprednisolone pulse therapy comprised 250-1000 mg of methylprednisolone that was intravenously administered for three consecutive days. Cyclophosphamide usage was given as a daily oral dose or intravenous therapy. Infectious complications were defined as severe conditions requiring antibiotic, antimycotic, and antiviral therapy to treat a bacterial, fungal, or viral infection, respectively. Steroid-induced diabetes mellitus was defined when insulin or a hypoglycemic agent needed to be administered after the initiation of the initial therapy.

Statistical analysis

Continuous variables are presented as median and range. Categorical variables are presented as number (n) and frequency (%). Continuous variables between the two groups were compared using the Mann-Whitney U-test while the categorical variables were compared using Fisher’s exact probability test. To determine the association between the types of initial immunosuppressive therapy and the development of infectious complications, we performed the logistic regression analyses. Accordingly, we evaluated the association between the development of infectious complications and the factors (age, history of diabetes mellitus, dose of PSL, rapid PSL reduction, methylprednisolone pulse, and cyclophosphamide therapy) that were thought to be associated with the incidence of infectious complications. In addition, we performed a multivariate logistic regression analysis adjusted for age and sex that are general confounding factors. P-value < 0.05 was considered to indicate statistical significance. Analyses were performed using EZR (Saitama Medical Center, Jichi Medical University, Saitama, Japan), which is a graphical user interface for R (The R Foundation for Statistical Computing, Vienna, Austria) [9].

## Results

Of the 47 patients, 21 (44.7%) developed infectious diseases requiring antibiotic, antimycotic, or antiviral therapy to treat a bacterial, fungal, or viral infection, respectively (Table 1). We compared clinical characteristics between patients with and without infectious diseases. Blood urea nitrogen, serum creatinine level, and frequency of proteinuria were significantly higher in patients with developing infectious complications than in those without (Table 1). In addition, the frequency of methylprednisolone pulse, cyclophosphamide therapy, end-stage renal disease, and/or death was higher in patients with developing infectious complications (Table 1). Two patients were treated with plasma exchange, and these two patients were treated with methylprednisolone and intravenous cyclophosphamide therapy (not shown in Table 1). No patients were treated with rituximab. However, age and lung complications, such as interstitial pneumonia and alveolar hemorrhage, and the frequency of treating with sulfamethoxazole-trimethoprim, were not significantly different between the patient group with and without developing infectious complications (Table 1).

**Table 1:** Comparison of clinical characteristics between patients with and without infectious complications Continuous variables are presented as median and range. Categorical variables are presented as number (n) and frequency (%). Categorical variables were compared using the Fisher exact probability test, and continuous variables were compared using the Mann-Whitney U-test. Significant differences are indicated with asterisks (* p <0.05). ANCA: anti-neutrophil cytoplasmic antibody; BMI: body mass index; CY: cyclophosphamide; DM: diabetes mellitus; ESRD: end stage renal disease; MPO: myeloperoxidase; mPSL: methylprednisolone; PR3: proteinase 3; PSL: prednisolone; ST: sulfamethoxazole-trimethoprim

	Infectious diseases		Infectious diseases		p value
	(+)		(-)		
	n = 21		n = 26		
Age	76	57-92	77	63-91	0.99
Male (n,%)	10	47.6	10	38.5	0.57
BMI (kg/m2)	23.4	18.5-30.8	21.7	15.8-28.8	0.50
Follow-up period (month)	20	1-67	22	1-73	0.72
History of DM (n,%)	4	19.0	5	19.2	1.00
History of hypertension (n,%)	10	47.6	12	46.2	1.00
MPO-ANCA (n,%)	20	95.2	20	76.9	0.11
PR3-ANCA (n,%)	2	9.6	9	34.6	0.08
Double positive (n,%)	1	4.8	3	11.5	0.62
Fever (n,%)	4	19.0	5	19.2	1.00
Lung complication					
Interstitial pneumonia (n,%)	11	52.4	11	42.3	0.56
Alveolar hemorrhage (n,%)	3	14.3	0	0	0.08
Neurological symptoms (n,%)	3	14.3	1	3.8	0.31
Skin lesion (n,%)	3	14.3	2	7.6	0.64
Systolic blood pressure (mmHg)	128	99-191	124	90-162	0.06
Diastolic blood pressure (mmHg)	72	57-108	68	40-96	0.09
Heart rate (/min)	80	54-105	82	60-101	0.71
Laboratory data					
Hemoglobin (g/dL)	10.3	6.3-13.9	9.7	5.1-15.3	0.98
Albumin (g/dL)	2.9	1.7-3.9	3	1.2-3.9	0.65
Blood urea nitrogen (mg/dL)	33.5	12.2-118.9	21.7	7.9-126.7	0.048*
Creatinine (mg/dl)	2.36	0.67-9.77	1.21	0.40-6.08	0.017*
C-reactive protein (mg/dl)	8.02	0.11-22.23	6.97	0.06-23.07	0.82
Hematuria (n,%)	19	90.5	17	65.4	0.08
Proteinuria (n,%)	20	95.2	16	61.5	0.013*
Treatment pattern					
PSL (maximum) (mg/kg/day)	0.66	0.36-1.01	0.62	0.00-1.21	0.78
Rapid PSL reduction (n,%)	7	33.3	15	57.7	0.14
mPSL pulse (n,%)	13	61.9	8	30.8	0.043*
CY therapy (n,%)	10	47.6	4	15.4	0.025*
ST mixture (n,%)	19	90.5	19	73.1	0.16
Prognosis					
ESRD (n,%)	7	33.3	2	7.6	0.058
All cause of death (n,%)	8	38.1	5	19.2	0.20
ESRD and/or death (n,%)	12	57.1	6	23.1	0.033*
Complications of treatment					
Steroid-induced DM (n,%)	7	33.3	3	11.5	0.09

Although age, sex, history of diabetes mellitus, lung complications, and maximum PSL dose were not significantly associated with developing infectious complications, serum creatinine level, methylprednisolone pulse, and cyclophosphamide therapy were significantly associated with developing infectious complications (Table 2).

**Table 2:** Univariate analysis of factors associated with the development of infectious complications Logistic regression analyses were performed to determine the factors associated with the development of infectious complications. Significant differences are indicated with asterisks (* p <0.05). CI: confidence interval; CY: cyclophosphamide; DM: diabetes mellitus; mPSL: methylprednisolone; OR: odds ratio; PSL: prednisolone

	Univariate analysis		
	OR	95%CI	p-value
Age	0.99	0.93-1.06	0.77
Male	1.45	0.45-4.66	0.53
History of DM	0.99	0.23-4.26	0.99
Lung complication	1.82	0.57-5.82	0.31
Serum creatinine	1.46	1.05-2.04	0.026*
PSL (mg/kg/day)	1.44	0.12-16.7	0.77
mPSL pulse	3.66	1.09-12.3	0.036*
CY therapy	5.00	1.27-19.6	0.021*

In addition, multivariate logistic regression analyses adjusted for age and sex revealed that serum creatinine level, methylprednisolone pulse, and cyclophosphamide therapy were also significantly associated with developing infectious complications (Table 3).

**Table 3:** Multivariate analysis of factors associated with the development of infectious complications Multiple logistic regression analyses were performed to determine the factors associated with the development of infectious complications. These factors were adjusted by age, and sex, respectively. Significant differences are indicated with asterisks (*p <0.05). CI: confidence interval; CY: cyclophosphamide; DM: diabetes mellitus; mPSL: methylprednisolone; OR: odds ratio; PSL: prednisolone

	Multivariate analysis		
	OR	95%CI	p-value
History of DM	1.04	0.24-4.54	0.96
Lung complication	1.79	0.53-6.01	0.35
Serum creatinine	1.46	1.04-2.05	0.027*
PSL (mg/kg/day)	1.46	0.10-21.5	0.78
mPSL pulse	4.85	1.09-21.5	0.038*
CY therapy	5.32	1.28-22.2	0.022*

**Figure 1:**
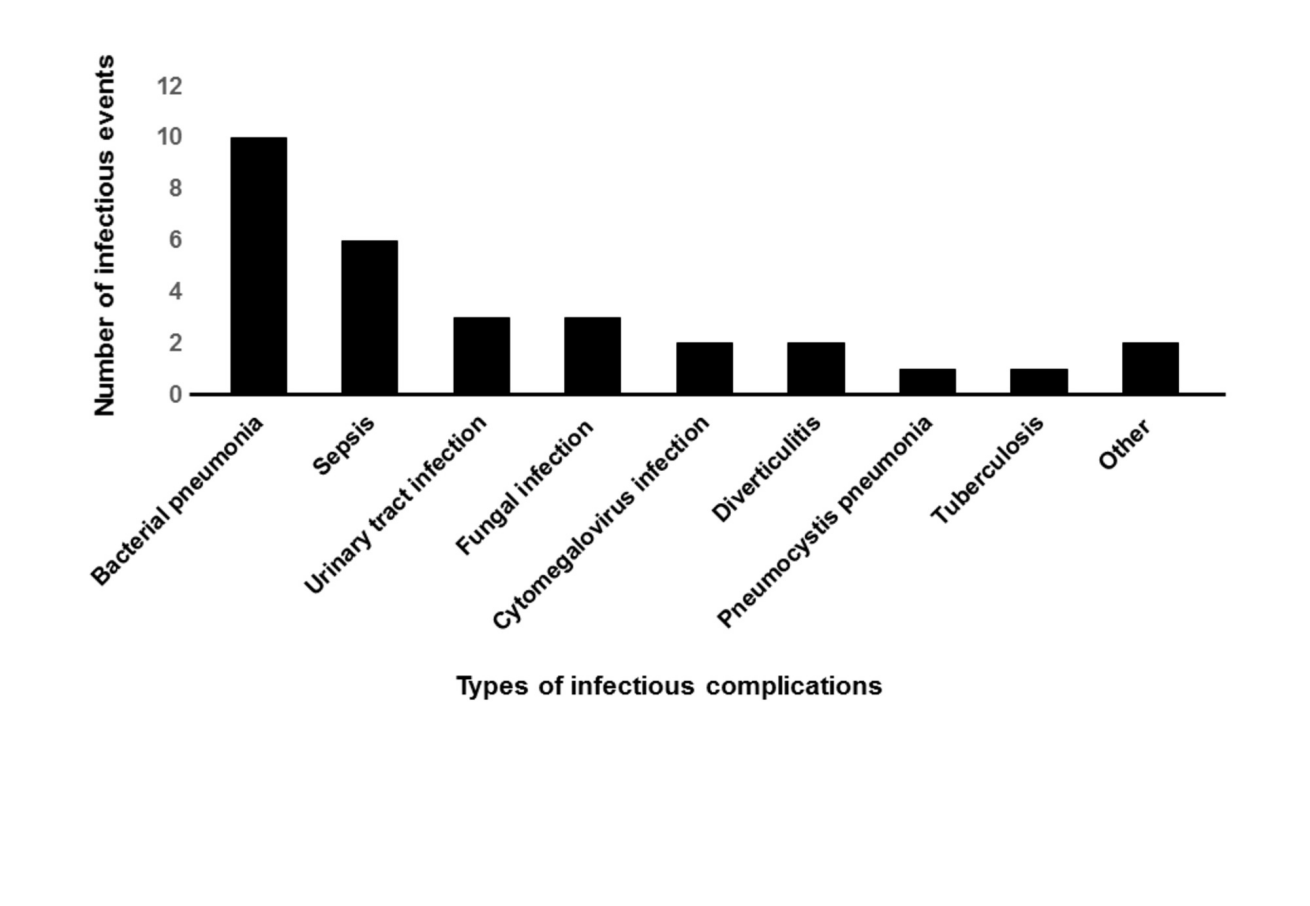
Types of each infectious event Because some patients suffered from multiple infectious events, the sum of the infectious events exceeded 21. Of these 21 patients, 10 developed bacterial pneumonia and six developed sepsis.

Figure 1 indicates the types of each infectious event, and because some patients suffered from multiple infectious events, the sum of the infectious events exceeded 21. Of these 21 patients, 10 developed bacterial pneumonia and six developed sepsis (Figure 1).

Types of causative bacteria of sepsis were methicillin-resistant Staphylococcus epidermidis, Escherichia coli, Bacteroides fragilis, Enterococcus, and Pseudomonas aeruginosa. Two cases of methicillin-resistant Staph epidermidis infection were catheter-related bloodstream infection (Table 4).

**Table 4:** Clinical characteristics of patients who developed infectious complications ANCA: anti-neutrophil cytoplasmic antibody; CMV: cytomegalovirus; CRBSI: catheter-related bloodstream infection; CY: cyclophosphamide; F: female; M: male; MPO: myeloperoxidase; mPSL: methylprednisolone; MRSE: methicillin-resistant Staphylococcus epidermidis; PR3: proteinase 3; PSL: prednisolone; UTI: urinary tract infection

Age	Sex	ANCA type	PSL	Rapid PSL	mPSL	CY	First infection from	Details of all infectious complications
			(mg/kg/day)	reduction			initiation of initial therapy therapy therapy	developed during the follow-up
68	M	PR3	0.81	-	+	+	4	Bacterial pneumonia
68	F	MPO	0.83	-	-	+	36	UTI and sepsis (Escherichia coli)
84	F	MPO	0.59	+	-	+	2	Pulmonary fungal infection (Aspergillus), CMV infection,
								UTI, and sepsis (Bacteroides fragilis and Enterococcus)
82	M	MPO, PR3	0.36	+	-	-	17	Herpes zoster and sepsis (MRSE)
90	F	MPO	0.39	+	-	-	3	Diverticulitis and peritonitis
57	M	MPO	0.75	-	+	-	12	Tuberculosis
75	F	MPO	0.59	-	-	-	1	Bacterial pneumonia, sepsis (CRBSI of MRSE), and
								systemic fungal infection (Cryptococcus neoformans)
92	F	MPO	0.44	-	+	-	1	Bacterial pneumonia
59	M	MPO	0.75	-	+	+	16	Diverticulitis
81	M	MPO	0.68	+	+	+	1	Pneumonia and sepsis (Pseudomonas aeruginosa)
78	F	MPO	0.92	-	+	+	2	UTI and bacterial pneumonia
74	M	MPO	1.00	-	+	-	1	Bacterial pneumonia
59	F	MPO	0.71	-	+	+	4	Bacterial pneumonia
79	F	MPO	0.57	-	-	-	8	Bacterial colitis
76	M	MPO	0.42	+	+	-	25	Tonsillitis
82	F	MPO	0.68	-	-	+	20	Pneumocystis pneumonia and CMV infection
68	M	MPO	0.65	-	+	+	20	Bacterial pneumonia
74	M	MPO	0.62	+	+	-	14	Bacterial pneumonia
72	F	MPO	0.66	-	-	-	5	Cryptococcal meningitis
79	M	MPO	0.97	-	+	-	1	Sepsis (CRBSI of MRSE)
78	F	MPO	0.53	+	+	+	3	Bacterial pneumonia

**Figure 2:**
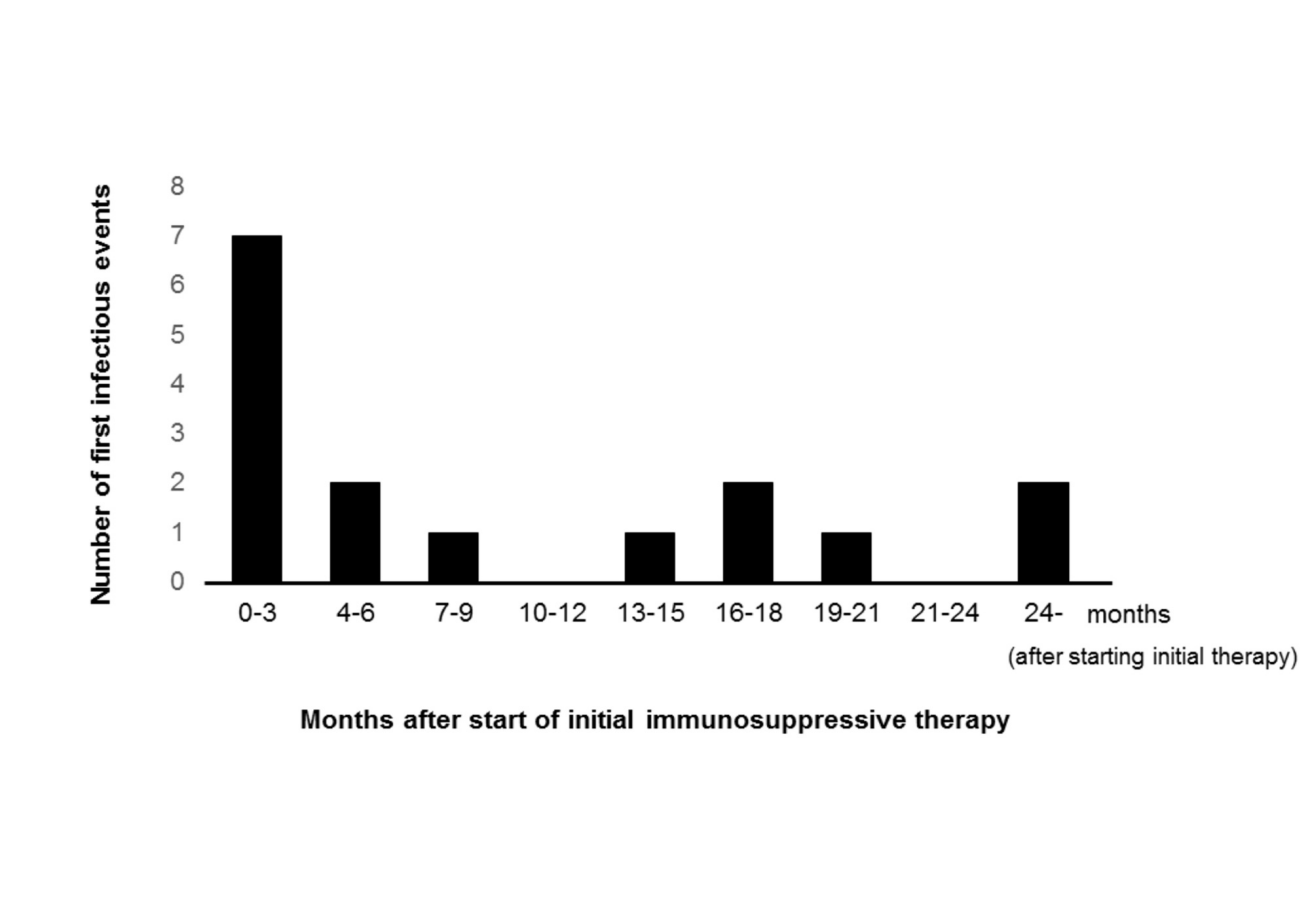
Timing of developing the first bacterial infectious complications Almost half of the patients developed infectious complications within six months from starting initial therapy. Because one patient developed multiple bacterial infections, the sum of the first bacterial infectious events exceeded 21.

In one case, two types of bacteria were detected at the same time. Regarding the timing of developing the first bacterial infectious complication, almost half of the patients developed infectious complications within six months from starting initial therapy (Figure 2).

Because one patient developed multiple bacterial infections, the sum of the first bacterial infectious events exceeded 21. In addition, there were three fungal infections, all of which developed within six months (Table 4).

## Discussion

In this study, patients with AAV had a high incidence of infectious complications during the therapeutic course. Kidney dysfunction, methylprednisolone pulse, and cyclophosphamide therapy were significantly associated with the development of infectious complications. It is believed that patients with severe kidney dysfunction were treated using strong immunosuppressive therapy (methylprednisolone pulse and/or cyclophosphamide therapy) because of which they often developed infectious complications. However, age, sex, history of diabetes mellitus, lung complications, and PSL dose were not associated. Age was not associated with the development of infectious complications. Although a previous study indicated that age tends to be associated with the development of infectious complications [8], it is believed that because most of the patients in the current study were elderly (the mean age was 75.4 ± 8.7 years), and age may not be significantly associated with the development of infectious diseases.

Regarding the types of infectious complications, bacterial pneumonia was the most common followed by sepsis. Urinary tract infection, fungal infection, and tuberculosis were also observed. Our study revealed that most of the patients developed their first infectious complication within six months of starting initial therapy. The risk of developing an infectious complication, including fungal infections, was the highest during the first six months.

PSL is mainly used for the treatment of AAV [1-2]. In Japan, most patients with AAV are treated with steroids and approximately 30% are treated with cyclophosphamide as initial therapy [2]. According to a previous nationwide study in Japan, the initial PSL dose was significantly associated with death and was gradually decreased annually [4,10]. The initial PSL dose of patients who were diagnosed with AAV from 1989-1998 was 0.85 mg/kg/day, whereas it was 0.71 mg/kg/day for those who were diagnosed after 2003 [4,10]. The mean initial PSL dose in the current study (0.65 mg/kg/day) was less than that for previous patient groups. The treatment algorithm for ANCA-associated rapid progressive glomerulonephritis (RPGN) recommends that the daily oral PSL dose should be reduced to <20 mg/day within four to eight weeks after the initiation of initial immune suppression therapy to prevent death from a subsequent infection [4]. However, in the current study, the initial PSL dose and PSL reduction to <20 mg/day were not significantly associated with the development of infectious complications. A possible reason was that our study had a short follow-up.

The treatment algorithm for ANCA-associated RPGN includes methylprednisolone pulse therapy [4]. Methylprednisolone pulse therapy has been administered in 44% of patients with MPA and 39% patients with GPA in Japan [2]. In the current study, 44.7% of patients underwent methylprednisolone therapy. Although there has been only one study that investigated the risk factors associated with treatment patterns in patients with AAV, a study by Watanabe-Imai et al. indicated that the initial dose of daily oral PSL but not of methylprednisolone pulse therapy was significantly associated with the development of infectious complications [8]. However, in patients with other autoimmune diseases, such as systemic lupus erythematosus (SLE), methylprednisolone pulse therapy was significantly associated with infectious complications [11]. Methylprednisolone pulse decreases B-cell lymphocyte function (B-cell proliferation and immunoglobulin synthesis) and T-cell response to inflammatory cytokine. These effects could last up to three months [11]. This may be one of the reasons why most infectious complications develop during the early phase of starting initial therapy. Cyclophosphamide has an important role in initial therapy for AAV [1-5]. A previous Japanese study indicated that 31% of patients with MPA and 60% of patients with GPA were treated with cyclophosphamide therapy [2]. A previous study on patients with SLE reported that sequential cyclophosphamide therapy was significantly associated with the development of infectious complications within three months [12]. As our study demonstrated, it is possible that cyclophosphamide therapy in patients with AAV may also be associated with the development of infectious complications within a few months of starting initial therapy. Thus, methylprednisolone pulse and cyclophosphamide therapy may be risk factors of infectious complications in the early phase (within six months) of AAV treatment.

With respect to maintenance therapy for AAV, of the 47 patients, although most patients were treated with PSL alone, three patients were treated with PSL and azathioprine and two were treated with PSL and mizoribine. Although the current study indicated that patients who received initial therapy within six months were at high risk of infectious complications, after a long time from initiation of initial therapy, the patterns of maintenance therapy may have more influence on the development of infectious complications. The current study did not include long-term follow-up of the enrolled patients. Further investigation will be needed.

With respect to AAV and incidences of infectious complications, a previous report suggested that severe infections occurred in 26%-46% of patients with GPA, and the leading cause of infection was pneumonia and sepsis followed [13]. Our result was almost similar to that of the previous study. Mohammad et al. also suggested that the incidence of severe infection was markedly higher in patients with AAV than in the background population [14]. Although approximately half of the infectious complications developed within six months, fungal infections also developed in the early phase after initiation of initial therapy (within six months). Cryptococcal bacteremia, meningitis, and pulmonary Aspergillus infection were observed in the current study (Table 4). Regarding Cryptococcal infection in patients with AAV, three case reports have been published [15-17] and are summarized in Table 5.

**Table 5:** Previous reports on Cryptococcus infection in patients with AAV AAV: ANCA associated vasculitis; ANCA: anti-neutrophil cytoplasmic antibody; CY: cyclophosphamide; F: female; M: male; MPO: myeloperoxidase; mPSL: methylprednisolone; PR3: proteinase 3; PSL: prednisolone

	Age	Sex	ANCA type	Clinical presentation of Cryptococcus	Treatment pattern	Timing of onset
[15]	46	F	PR3	meningitis, pneumonia, and bacteremia	PSL, mPSL, and CY	2 months after AAV relapse
[16]	67	M	MPO	meningitis and pneumonia	PSL, and mPSL	5 months after AAV relapse
[17]	81	F	MPO	meningitis	PSL	2 months after the initial therapy

All three patients in these cases developed Cryptococcal meningitis, and C. neoformans was isolated from the cerebrospinal fluid. Two cases developed after the relapse of AAV and initiation of induction therapy and one developed within two months from initiation of initial immune suppressive therapy. Regarding Aspergillus infection in patients with AAV, five case reports and one clinical study have been reported previously [18-23]. The case reports are summarized in Table 6.

**Table 6:** Previous reports on Aspergillus infection in patients with AAV AAV: ANCA-associated vasculitis; ANCA: anti-neutrophil cytoplasmic antibody; CY: cyclophosphamide; F: female; M: male; MPO: myeloperoxidase; mPSL: methylprednisolone; PR3: proteinase 3; PSL: prednisolone

	Age	Sex	ANCA type	Clinical presentation of Aspergillus	Treatment pattern	Timing of onset
[18]	48	M	PR3	pulmonary aspergillosis	PSL and CY	5 months after the initial therapy
[19]	47	F	MPO	chronic necrotizing pulmonary aspergillosis	PSL and mPSL	3 months after the initial therapy
[20]	77	F	MPO	invasive pulmonary aspergillosis	PSL and mPSL	3 months after the initial therapy
[21]	71	M	MPO	pulmonary aspergilloma	PSL and mPSL	11 days after the initial therapy
[22]	75	M	no data	pleuritis	PSL and CY	22 months after the initial therapy

All of these cases were reported from Japan, and all patients were treated with PSL and methylprednisolone pulse or cyclophosphamide therapy. Four of the five patients developed Aspergillus infection within six months from the initiation of initial therapy (Table 6). One clinical research study reported by Su et al. stated that seven of 157 patients with AAV developed invasive pulmonary aspergillosis [23]. Of these seven cases of invasive pulmonary aspergillosis, two were GPA and five were MPA. All cases of invasive pulmonary aspergillosis developed within two to 13 weeks from the initiation of initial immune suppressive therapy [23]. Therefore, we should also consider the possibility of these fungal pathogens as well as common bacterial infections in patients with suspected infectious complications. We suspect that the incidence of infectious complications was high in the early phase (within six months) of AAV treatment because the amount of immune suppression agent was high in the early phase (within six months) after the initiation of initial therapy.

Concerning the analyses of death cases, of 13 death cases, five patients died of infectious complication (there was sepsis, one had peritonitis, and one had pneumocystis pneumonia) (Table 7). In addition, four patients died of AAV-related complication and another four patients died of cardiovascular diseases (Table 7).

**Table 7:** Clinical data of the death cases AAV: anti-neutrophil cytoplasmic antibody associated vasculitis; CY: cyclophosphamide; F: female; M: male; mPSL: methylprednisolone; PCP: pneumocystis pneumonia; PSL: prednisolone; SAH: subarachnoid hemorrhage

Age	Sex	Renal	Respiratory	Cause of death	mPSL	CY
		Complication	Complication			
84	F	+	-	Infection (sepsis)	-	+
82	M	+	+	Infection (sepsis)	-	-
90	F	+	+	Infection (peritonitis)	-	-
75	F	+	+	Infection (sepsis)	-	-
82	F	+	+	Infection (PCP)	-	+
67	M	+	-	AAV related (SAH)	+	-
74	M	+	+	AAV related (interstitial pneumonia)	+	-
77	M	+	+	AAV related (alveolar hemorrhage)	+	-
91	F	+	+	AAV related (alveolar hemorrhage)	-	+
73	F	+	+	Cardiac (heart failure)	-	-
87	F	+	-	Cardiac (heart failure)	-	-
92	F	+	-	Cardiac (heart failure)	+	-
76	M	+	-	Cardiac (arrhythmia)	+	-

Some limitations of this study need to be acknowledged. This was a single-center retrospective study. However, the patients’ backgrounds were similar to those reported in a previous multicenter study in Japan. Because AAV is a rare disease, the number of patients in our study was small. This study also had a short follow-up, but this was because most Japanese patients with AAV are elderly. The choice and strength of the treatment pattern were at the discretion of each clinical physician. Although it is thought that most clinical physicians treat AAV based on the Japanese guidelines for AAV, there was no common treatment pattern in this study. Moreover, because this was a retrospective observational study, we could not completely evaluate the disease activity of AAV, particularly the Birmingham Vasculitis Activity Score. However, we evaluated fever, renal function, lung complication, skin lesion, and neurological findings. In addition, there were not enough data regarding the levels of immunoglobulins, complements, and nutritional factors other than serum albumin. Further, this study focused on the details and risks of infectious complications. Therefore, we cannot determine whether we should treat AAV patients with methylprednisolone pulse and/or cyclophosphamide or not. Further investigation is required to elucidate the clinical efficiency and risk associated with methylprednisolone and/or cyclophosphamide therapy in the therapeutic course of patients with AAV. In addition, because rituximab therapy is not common among Japanese patients with AAV yet, the relationships between rituximab therapy and the development of infectious complications should be investigated in the future. Although the leading cause of infectious complications was bacterial pneumonia, we have no data regarding vaccination for streptococcal pneumonia, smoking, the complication of chronic obstructive lung disease, and human immunodeficiency virus (HIV) infection. These factors may also influence the development of infectious complications. As for the timing of the onset of infectious complication, route of administration (daily oral or intravenous) of cyclophosphamide may be associated. However, in the current study, of 14 patients treated with cyclophosphamide therapy, only one patient was treated with daily oral administration of cyclophosphamide.

## Conclusions

In the current study, we retrospectively investigated 47 newly diagnosed patients with AAV (patients with eosinophilic granulomatous polyangiitis were excluded). We determined the association between the types of initial immunosuppressive therapy and the development of infectious complications. In addition, we investigated the details of infectious complications, the timing of onset; and the types. Both methylprednisolone pulse and cyclophosphamide therapy are significantly associated with the development of infectious complications. Although bacterial pneumonia and sepsis often develop, a fungal infection may also develop in patients with AAV, particularly within three to six months after the initiation of treatment. Thus, information obtained from the current study is useful for early diagnosis and treatment against various infectious complications, including bacterial and fungal infection, in the therapeutic course of patients with AAV.
